# Supplementary material for: Gas Permeation of Sulfur Thin-Films and Potential as a Barrier Material
Source: Membranes (Basel). 2019 Jun 14;9(6):72. doi: 10.3390/membranes9060072 (PMC6631778; doi:10.3390/membranes9060072)
Supplement: Supplementary file 1 [file membranes-09-00072-s001.pdf]

membranes

Supporting Information: Gas permeation of sulfur thin-films and potential as a barrier material

Xicheng Jia [1], Thomas D. Bennett [2,*], and Matthew G. Cowan [1,*]

[1] Department of Chemical and Process Engineering, University of Canterbury, Christchurch, New Zealand
[2] Department of Materials Science and Metallurgy, University of Cambridge, Cambridge, CB3 0FS, UK.
* Correspondence: matthew.cowan@canterbury.ac.nz; Tdb35@cam.ac.uk

Received: date; Accepted: date; Published: date

Table of Contents

S1. Contextualization of novelty statements for the major areas of novelty identified in this work ... 2
S1.1 Contextualization of novelty statement for sulfur-derived polymers ... 2
S1.2 Contextualization of novelty statement for barrier materials ... 5
S1.3 Contextualization of Novelty Statement for sulfur mermbrane materials ... 6
S2. Experimental Section ... 9
S2.1 Thermogravimetric analysis (TGA) ... 9
S2.2 Differential Thermal Analysis (DTA) ... 10
S2.3 PXRD data of elemental sulfur (control) and sulfur membranes ... 11
References ... 12

S1. Contextualization of novelty statements for the major areas of novelty identified in this work

The following sections describe the search methodology used to contextualize the novelty of our proposed research and understand the surrounding science in the areas of sulfur-derived polymers (ESI Section S2.1) and sulfur-based barrier materials (ESI Section S2.2). In all cases, a selection of relevant references have been included in the main manuscript and full research notes can be obtained from the authors upon request.

S1.1 Contextualization of novelty statement for sulfur-derived polymers

Key idea for contextualization:
Scope of academic research on sulfur-derived polymeric materials.
Subject area(s):
Sulfur and polymer chemistry
Selected database(s) or search method and date of search:
Google scholar; 1 April 2019
Initial keyword(s) / Search String(s) / Chemical Structure(s)
Sulfur polymers
Number of search results and refinement string(s):
About 1,810,000
Final number of results to be analysed:
First 10 pages of results; 100 references
Explanation of how results were analysed:
Skim read the titles and looked at citation count to access the impact of the work. Read a small selection of papers seeking to identify prominent authors and key research in the field.
Summary of results (if not included in the publication):
Came across the key term of 'inverse vulcanization.' Further investigation of the area identified the paper "The use of elemental sulfur as an alternative feedstock for polymeric material" as a seminal work in the area.[1]
Were additional keywords / search methods identified:
Yes – see additional CON statement(s)
~~No – search concluded~~
Time spent: 1 hour

Key idea for contextualization:
Scope of academic research on sulfur-derived polymeric materials.
Subject area(s):
Sulfur and polymer chemistry
Selected database(s) or search method and date of search:
Google scholar; 1 April 2019
Initial keyword(s) / Search String(s) / Chemical Structure(s)
The use of elemental sulfur as an alternative feedstock for polymeric materials
Number of search results and refinement string(s):
391
Final number of results to be analysed:
391
Explanation of how results were analysed:
The titles and first three lines of the abstracts as provided by google scholar were read. Papers of particular interest, especially review papers were selected and read in depth.
Summary of results (if not included in the publication):
A range of applications and review articles were identified, providing background reading for the project. No evidence of references associated with the study of gas permeation through these materials was identified. Several key reviews were selected for further study including:<br>“Recent Approaches for the Direct Use of Elemental Sulfur in the Synthesis and Processing of Advanced Materials”[2]<br>“Sulfur and Its Role In Modern Materials Science”[3]<br>“Green chemistry and polymers made from sulfur”[4]<br>Citations of these papers were selected for examination.
Were additional keywords / search methods identified:
Yes – see additional CON statement(s)
~~No – search concluded~~
Time spent: 5 hours

Key idea for contextualization:
Scope of academic research on sulfur-derived polymeric materials.
Subject area(s):
Sulfur and polymer chemistry
Selected database(s) or search method and date of search:
Google scholar; 4-8 April 2019
Initial keyword(s) / Search String(s) / Chemical Structure(s)
"Recent Approaches for the Direct Use of Elemental Sulfur in the Synthesis and Processing of Advanced Materials"<br>"Sulfur and Its Role In Modern Materials Science"<br>"Green chemistry and polymers made from sulfur"
Number of search results and refinement string(s):
Respectively:<br>94 results<br>76 results<br>39 results
Final number of results to be analysed:
209
Explanation of how results were analysed:
The titles and first three lines of the abstracts as provided by google scholar were read. Papers of particular interest, especially review papers were selected and read in depth.
Summary of results (if not included in the publication):
A range of applications and review articles were identified, providing background reading for the project. No evidence of references associated with the study of gas permeation through these materials was identified.<br>Judged at this point a sufficient overview of the field had been obtained.
Were additional keywords / search methods identified:
~~Yes – see additional CON statement(s)~~
No – search concluded
Time spent: 3 days

S1.2 Contextualization of novelty statement for barrier materials

Key topic for contextualization:
Understanding of barrier materials, desirable features, and gas permeability of common materials
Subject area(s):
Barrier materials
Selected database(s) or search method and date of search:
Google scholar; 1 May 2019
Initial keyword(s) / Search String(s) / Chemical Structure(s)
permeance of barrier materials
Number of search results and refinement string(s):
About 13,900
Final number of results to be analysed:
Ca. 100
Explanation of how results were analysed:
The titles and abstracts were scanned, particularly for review articles.
Summary of results (if not included in the publication):
Key works identified:<br>“Plastics Design Library : Permeability Properties of Plastics and Elastomers :<br>A Guideto Packaging and Barrier Materials (2nd Edition)” [5] Contained extensive information on barrier materials and standard test methods.<br>Subsequent searches easily identified permeability data on common barrier materials under exposure for a range of common materials. Cellophane and PVDC were selected for comparison.
Were additional keywords / search methods identified:
~~Yes – see additional CON statement(s)~~
No – search concluded
Time spent: 8 hours

S1.3 Contextualization of Novelty Statement for sulfur mermbrane materials

Key Novelty Claim(s):
Sulfur membranes have not been characterized for gas permeability.
Subject area(s):
Membrane science
Selected database(s) or search method and date of search:
Scifinder; 29 May 2019
Initial keyword(s) / Search String(s) / Chemical Structure(s)
Sulfur membrane
Number of search results and refinement string(s):
30
Final number of results to be analysed:
30
Explanation of how results were analysed:
The titles and abstracts were read.
Summary of results (if not included in the publication):
No relevant papers were identified.<br>The closest was a paper on a Li_2S containing composite used as a battery electrode.
Were additional keywords / search methods identified:
Yes – see additional CON statement(s)
~~No – search concluded~~
Time spent: 1 hour

Key Novelty Claim(s):
Sulfur membranes have not been characterized for gas permeability.
Subject area(s):
Membrane science
Selected database(s) or search method and date of search:
Google scholar; last repeated 29 May 2019
Initial keyword(s) / Search String(s) / Chemical Structure(s)
sulfur-based membrane gas separation
Number of search results and refinement string(s):
About 4500
Final number of results to be analysed:
200
Explanation of how results were analysed:
The titles and abstracts were read of papers in the first 20 pages of results.
Summary of results (if not included in the publication):
One relevant paper was identified.<br>"Direct Utilization of Elemental Sulfur in the Synthesis of Microporous Polymers for Natural Gas Sweetening"[6]
Were additional keywords / search methods identified:
Yes – see additional CON statement(s)
~~No – search concluded~~
Time spent: 1 hour

Key Novelty Claim(s):
Sulfur membranes have not been characterized for gas permeability.
Subject area(s):
Membrane science
Selected database(s) or search method and date of search:
Google scholar; last repeated 29 May 2019
Initial keyword(s) / Search String(s) / Chemical Structure(s)
Elemental sulfur membrane gas separation
Number of search results and refinement string(s):
About 48300
Final number of results to be analysed:
200
Explanation of how results were analysed:
The titles and abstracts were read of papers in the first 20 pages of results.
Summary of results (if not included in the publication):
One relevant paper was identified.<br>"Direct Utilization of Elemental Sulfur in the Synthesis of Microporous Polymers for Natural Gas Sweetening"[6]
Were additional keywords / search methods identified:
~~Yes – see additional CON statement(s)~~
No – search concluded
Time spent: 1 hour

S2. Experimental Section

S2.1 Thermogravimetric analysis (TGA)

One TGA study was undertaken on sulfur powder (S_8) to check the phase transition under N_2 atmosphere over a period time of 5 h. To summarize, the TGA results show that S_8 didn't show obvious mass loss until 200 °C, and the mass loss after 200 °C is due to sublimation. The temperature profile used was equilibrate to 50 °C, then ramped to 300 °C at 10 °C/min under N_2 environment.

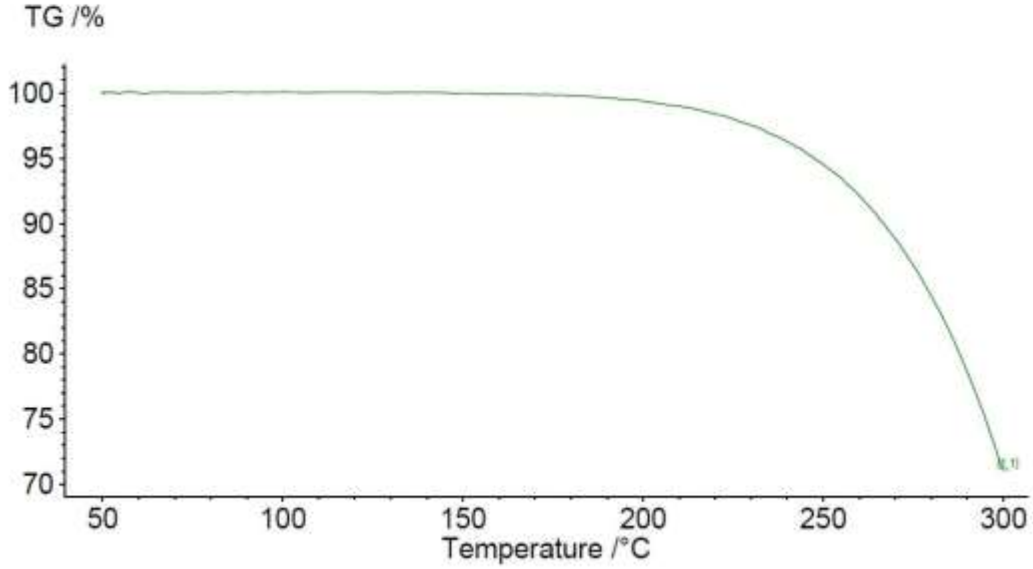

Fig S1. TGA data for sulfur powder (S_8) using the mass at 50 °C as the reference for the relative mass calculation.

S2.2 Differential Thermal Analysis (DTA)

One DTA study was undertaken on sulfur powder (S_8) to check the phase transition under N_2 atmosphere over a period time of 5 h. To summarize, the DTA results shown that S_8 experienced 3 phase transitions during the testing time. The phase transition was happened at 103.8 °C, 109.6 °C and 172.4 °C. The membrane was hence processed at temperatures above the third phase transition point, i.e., 175 °C, due to the fast mobility and lower viscosity of the melt observed at this temperature.

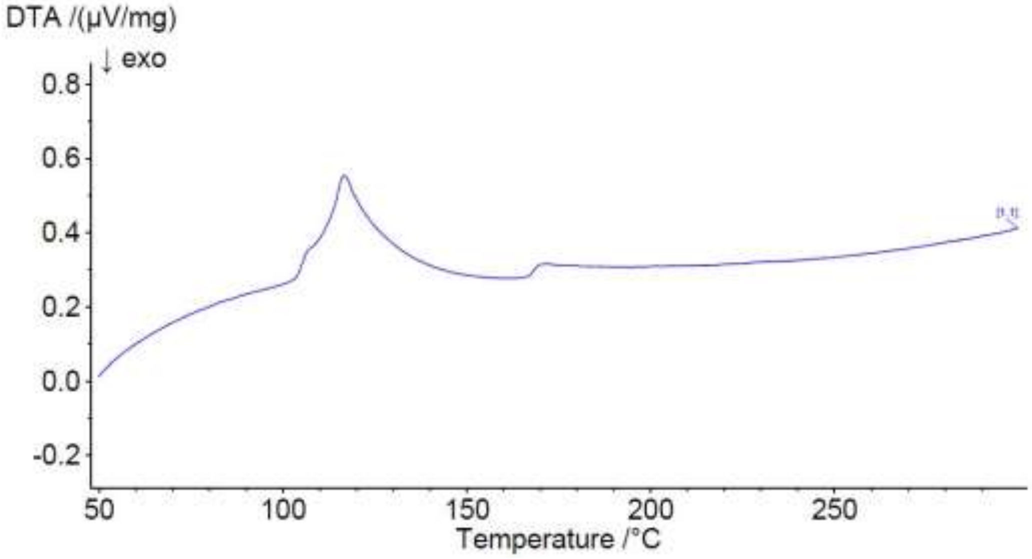

Fig S2. DTA data for sulfur powder (S_8) during the first cycle.

S2.3 PXRD data of elemental sulfur (control) and sulfur membranes

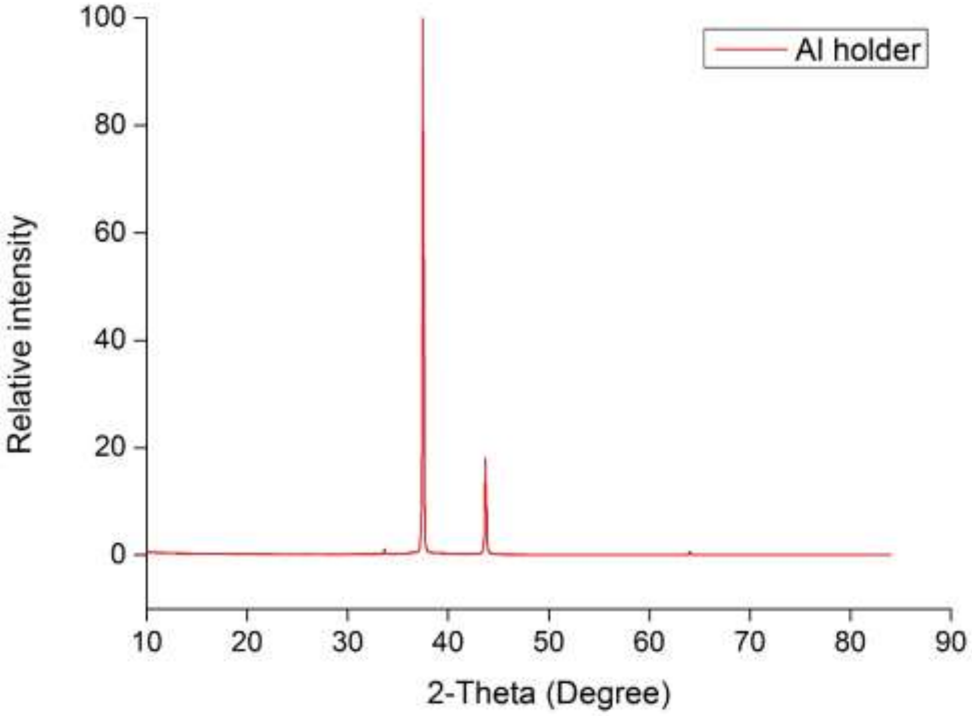

Fig S3. PXRD of aluminium sample holder.

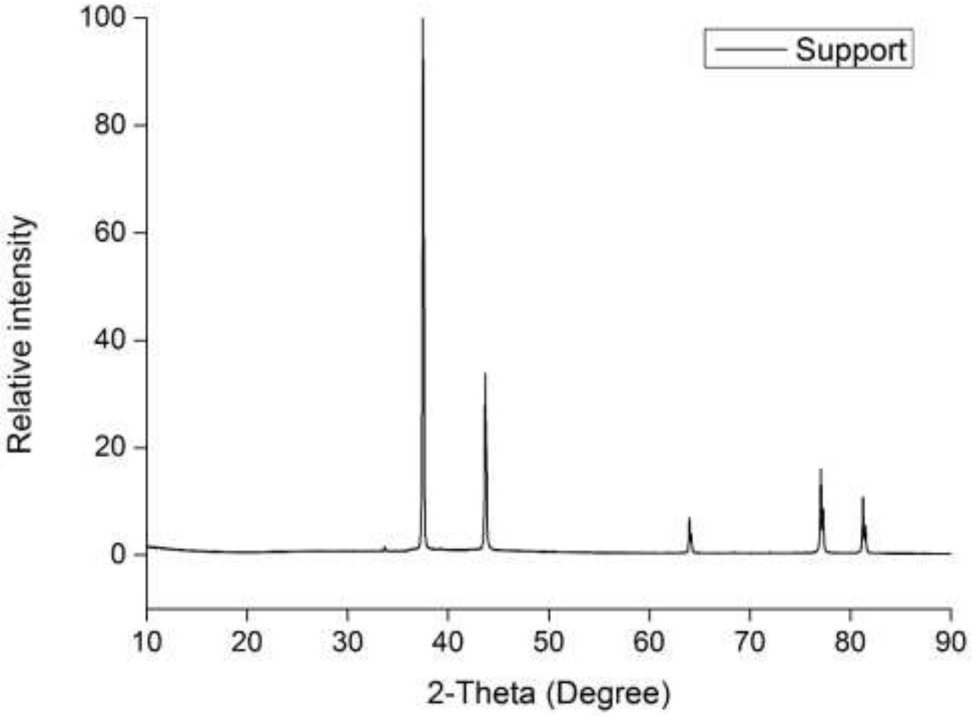

Fig S4. PXRD of PES support.

References

1. Chung, W. J.; Griebel, J. J.; Kim, E. T.; Yoon, H.; Simmonds, A. G.; Ji, H. J.; Dirlam, P. T.; Glass, R. S.; Wie, J. J.; Nguyen, N. A., The use of elemental sulfur as an alternative feedstock for polymeric materials. *Nature chemistry* **2013,** *5* (6), 518.
2. Lim, J.; Pyun, J.; Char, K., Recent approaches for the direct use of elemental sulfur in the synthesis and processing of advanced materials. *Angewandte Chemie International Edition* **2015,** *54* (11), 3249-3258.
3. Boyd, D. A., Sulfur and its role in modern materials science. *Angewandte Chemie International Edition* **2016,** *55* (50), 15486-15502.
4. Worthington, M. J.; Kucera, R. L.; Chalker, J. M., Green chemistry and polymers made from sulfur. *Green Chemistry* **2017,** *19* (12), 2748-2761.
5. Massey, L. K., Introduction. In *Permeability Properties of Plastics and Elastomers (Second Edition)*, Massey, L. K., Ed. William Andrew Publishing: Norwich, NY, 2003; pp 1-56.
6. Je, Sang H.; Buyukcakir, O.; Kim, D.; Coskun, A., Direct Utilization of Elemental Sulfur in the Synthesis of Microporous Polymers for Natural Gas Sweetening. *Chem* **2016,** *1* (3), 482-493.
